# The lncRNA XIST interacts with miR-140/miR-124/iASPP axis to promote pancreatic carcinoma growth

**DOI:** 10.18632/oncotarget.22555

**Published:** 2017-11-20

**Authors:** Shuai Liang, Xuejun Gong, Gewen Zhang, Gengwen Huang, Yebin Lu, Yixiong Li

**Affiliations:** ^1^ Department of Pancreatic Biliary Surgery, Xiangya Hospital, Central South University, Changsha 410008, China

**Keywords:** XIST, pancreatic carcinoma (PC), cell cycle, miR-140/miR-124, iASPP

## Abstract

Long non-coding RNA (lncRNA) X-inactive specific transcript (XIST) is involved in the development and progression of many tumors. In this study, XIST was specifically upregulated in pancreatic carcinoma tissues and cell lines; a higher XIST expression was correlated to poorer clinicopathologic features. After XIST knockdown, the proliferation of PC cell lines was suppressed and cell cycle stagnated in G1 phase; XIST knockdown also reduced the protein levels of inhibitor of apoptosis-stimulating protein of p53 (iASPP) and Cyclin-dependent kinase 1 (CDK1), increased the protein level of P21, a potent CDK inhibitor. In PC cell lines, XIST and miR-140/miR-124, two tumor-associated miRNAs, could inversely regulate each other, respectively; miR-140/miR-124 could bind to XIST and the 3’UTR of *PPP1R13L*, respectively. XIST and miR-140/miR-124 exerted opposite effects on iASPP, CDK1, P21 and P27 proteins; whereas the effects of LV-sh-XIST on the indicated protein levels could be partially reversed by miR-140 and/or miR-124 inhibitor. In PC tissues, miR-140 and miR-124 expression was down-regulated, iASPP and CDK1 mRNA expression was up-regulated. XIST positively correlated with iASPP and CDK1, inversely correlated with miR-140 and miR-124, respectively. Taken together, our data indicated that XIST might be an oncogenic lncRNA that promoted proliferation of PC cell line through inhibiting miR-140/miR-124 expression and promoting cell cycle-related factor expression, and could be regarded as a therapeutic target in human pancreatic carcinoma.

## INTRODUCTION

Pancreatic adenocarcinoma is a highly malignant phenotype characterized by rapid progression, early metastasis, and a limited response to radiotherapy and chemotherapy. In the past 10 years, despite of FDA approved therapeutic regimens and great improvements in medical care, we observed no momentous effect on PC patient survival [1, 2]. Therefore, progress in the treatment of PC now depends to a great extent on an increased understanding of the underlying molecular mechanisms and the development of innovation clinical approaches.

Human genome sequence data indicate that more than 90% of the DNA sequences actively transcribed, but only 2% of it encodes protein, thus the majority of transcripts are referred to as non-coding RNAs (ncRNAs) [3, 4]. Small non-coding RNAs such as microRNAs have been studied extensively and their roles in gene regulation and cell function have been elucidated innumerous cancers [4]. Recent studies have shown that lncRNAs play important roles in both normal development and diseases including cancer [5]. LncRNAs have emerged as new players in cancer research and several studies have shown that some lncRNAs function as oncogenes, tumor suppressor genes or both, depending on the circumstance [6]. In the discovered lncRNAs, X-inactive specific transcript (XIST) is involved in the development and progression of many tumors. XIST expression has been found to be dysregulated in a variety of human cancers when compared to normal cells; meanwhile, the dysregulation of XIST expression can also affect cancer cell proliferation, invasion and migration [7].

The mechanisms by which lncRNAs exert their effect varies under different conditions, however, emerging evidences have revealed that the interaction between lncRNAs and microRNAs plays a major role [8, 9]. Zhu et al. reported that lncRNA H19/miR-675 axis represses prostate cancer metastasis by targeting transforming growth factor beta-induced (TGFBI) [10]. The interaction between miR-141 and lncRNA-H19 has been regarded as an important component in regulating cell proliferation and migration in gastric cancer [11].

Inhibitor of apoptosis-stimulating protein of p53 (iASPP) is an evolutionally conserved inhibitory member of the ASPP protein family that can specifically inhibit the p53-mediated cell death [12]. Overexpression of iASPP was observed in several kinds of human tumors [12–15], suggesting that iASPP plays an important role in tumorigenesis. More importantly, iASPP and miRNAs work together to participate in tumorigenesis. Thus, iASPP might be a potential molecular target for cancer therapy.

In this study, we report an inverse dual regulation between X-inactive specific transcript (XIST) and miR-140/miR-124 which regulates PC cell proliferation and cell cycle through directly targeting iASPP. Our findings provide a novel understanding of the role of XIST in PC cell proliferation and the mechanism involved.

## RESULTS

### XIST is highly expressed in PC tissues and cell line and related to clinicopathologic features

**Figure 1:**
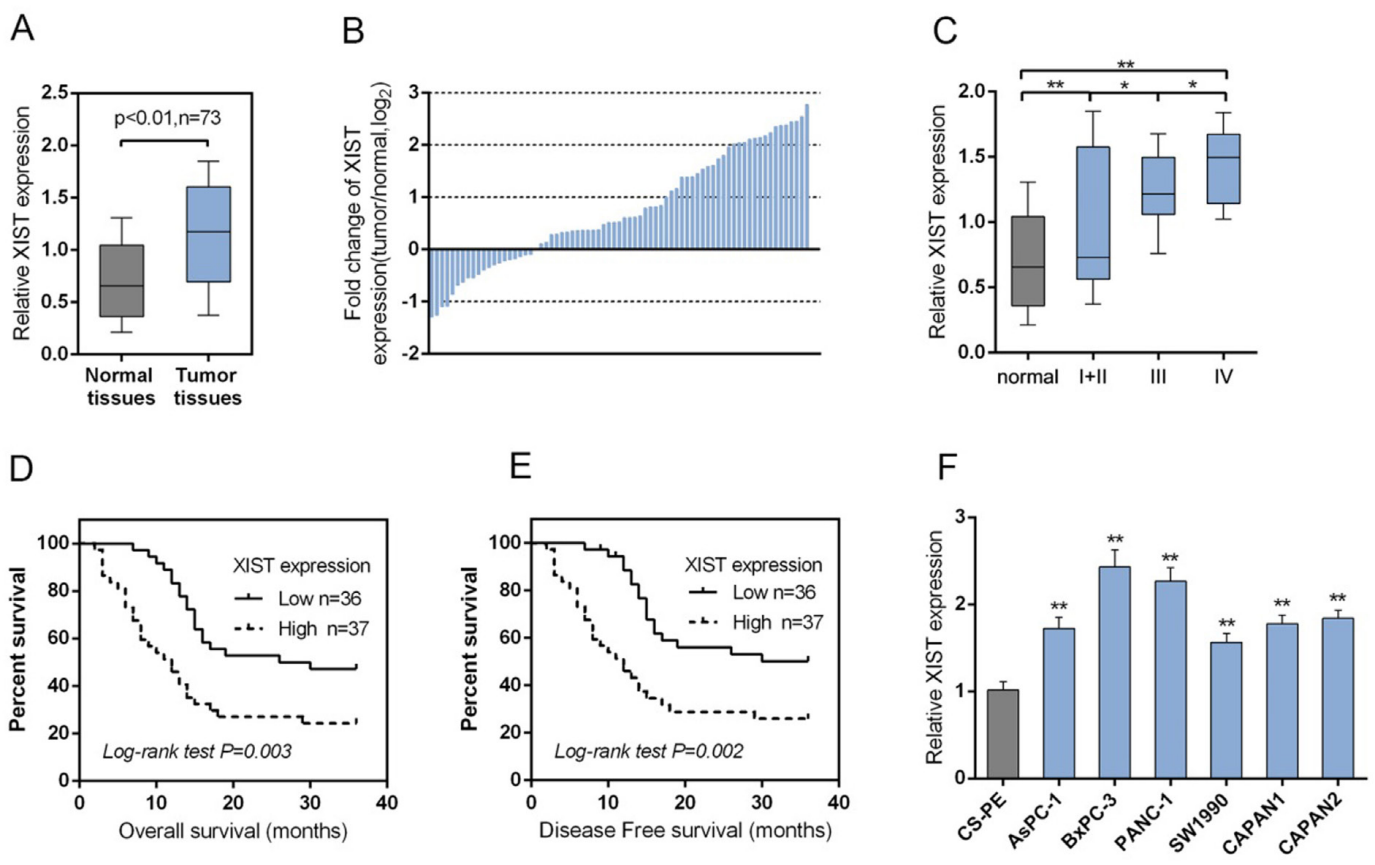
XIST is highly expressed in PC tissues and cell line and related with clinicopathologic features **(A)** A large panel of 73 paired PC tissues and matched adjacent normal tissues were obtained. The expression of XIST was monitored using SYBR green quantitative PCR. **(B)** Expression of XIST in 73 pairs of PC tissues and their corresponding adjacent non-tumorous tissues (ANTs) in a training cohort. Expression level of XIST was determined by real-time PCR and normalized to U6. Fold change were analyzed using the formula 2-(ΔΔCT [PC/ANT]). Red line indicates fold change of XIST equal to 2. **(C)** XIST expression in PC tissues of different TNM stages (I+II, III, IV) and adjacent normal tissues were determined by using real-time PCR assays. **(D)** The overall survival of PC patients with low XIST expression and high XIST expression. **(E)** The disease free survival of PC patients with low XIST expression and high XIST expression. **(F)** The expression of XIST in human pancreatic epithelial cell (CS-PE) and six human PC cell lines, including AsPC-1, BxPC3, SW1990, PANC-1, CAPAN-1 and CAPAN-2 was determined using real-time PCR. The data are shown as mean± SD of three independent experiments. ^*^P<0.05, ^**^P<0.01.

A large panel of 73 paired primary PC tissues and the matched adjacent normal tissues were obtained, and the expression of XIST was monitored. A significantly higher expression of XIST was observed in PC tissues, compared with that of the matched adjacent normal tissues (Figure 1A). To validate this result, we performed quantitative real-time PCR in 73 cases of PC tissues and adjacent normal tissues in training cohort. Compared to the corresponding normal tissues, XIST showed to be upregulated in 53 PC cases (> 72.6%), among them 27 PC cases (> 36.9%) were upregulated to more than 2-fold [i.e., log_2_ (fold change) > 1]) (Figure 1B). XIST expression was upregulated in PC tissues of I+II, III or IV TNM stage, compared with normal tissues (Figure 1C). 73 cases of PC tissues were divided into two groups: a high XIST expression group (above the median XIST expression, n = 37) and a low XIST expression group (below the median XIST expression, n = 36). High expression of XIST in PC showed to be related to the advanced TNM stage and larger tumor dimension according to clinicopathological parameters as exhibited in Table 1. To determine the potential relationship between XIST expression and the patients’ prognosis, Kaplan-Meier analysis and log-rank test was used to evaluate the effects of XIST expression on overall survival (OS) and disease free survival (DFS). The results indicated that patients with higher XIST expression had a significantly shorter OS (*P*=0.003) and DFS (*P*=0.002) compared to patients with lower XIST expression (Figure 1D and 1E). We also used the COX proportional hazard model to perform univariate and multivariate analysis of factors related to overall survival. As shown in Table 2, XIST showed to be an independent prognostic factor for pancreatic cancer. Then the XIST expression in cell lines was determined. XIST expressed at a higher level in six PC cell lines, AsPC-1, BxPC3, SW1990, PANC-1, CAPAN-1 and CAPAN-2, compared with that of in the normal cell line, the CS-PE (Figure 1F). These data suggested that XIST is highly expressed in PC tissues and cell lines, and is related to poorer clinicopathological parameters.

**Table 1:** Correlation of the expression of XIST with clinicopathologic features

Variables		XIST expression		P
		High	Low	
Age		45.65±13.38	42.36±18.22	0.459
Gender	female	18	16	0.719
	male	19	20	
Lymph node metastasis	No	17	21	0.290
	Yes	20	15	
Distant metastasis	No	18	19	0.724
	Yes	19	17	
TNM stage	I + II	13	27	0.003
	III	11	5	
	IV	13	4	
Largest tumor dimension	< 3	9	24	<0.001
	≥ 3	28	12	
Lymphatic invasion	No	22	18	0.417
	Yes	15	18	
Vascular invasion	No	15	20	0.199
	Yes	22	16	
Perineural invasion	No	20	17	0.559
	Yes	17	19	

**Table 2:** Univariate and multivariate analysis for factors related to overall survival using the COX proportional hazard model

Variables		Univariate analysis			Multivariate analysis		
		P	HR	95%CI	P	HR	95%CI
Age	44.027±15.926	0.470	1.007	0.989-1.025	N.A		
Gender	female vs male	0.477	1.231	0.694-2.181	N.A		
Lymph node metastasis	No vs Yes	0.330	0.752	0.424-1.335	N.A		
Distant metastasis	No vs Yes	0.767	1.090	0.615-1.934	N.A		
TNM stage	I + II vs III + IV	0.007	0.397	0.202-0.779	0.184	0.622	0.309-1.253
Largest tumor dimension	<3 vs ≥3	0.156	0.658	0.368-1.174	N.A		
Lymphatic invasion	No vs Yes	0.204	1.460	0.814-2.617	N.A		
Vascular invasion	No vs Yes	0.878	0.956	0.540-1.695	N.A		
Perineural invasion	No vs Yes	0.797	1.078	0.608-1.911	N.A		
XIST expression	high vs low	<0.001	3.727	1.964-7.072	0.001	3.336	1.686-6.601

### Knockdown of XIST induces cell cycle arrest at G0/G1 phase to suppress PC cell proliferation by regulating cell cycle-related genes in PC cell lines

**Figure 2:**
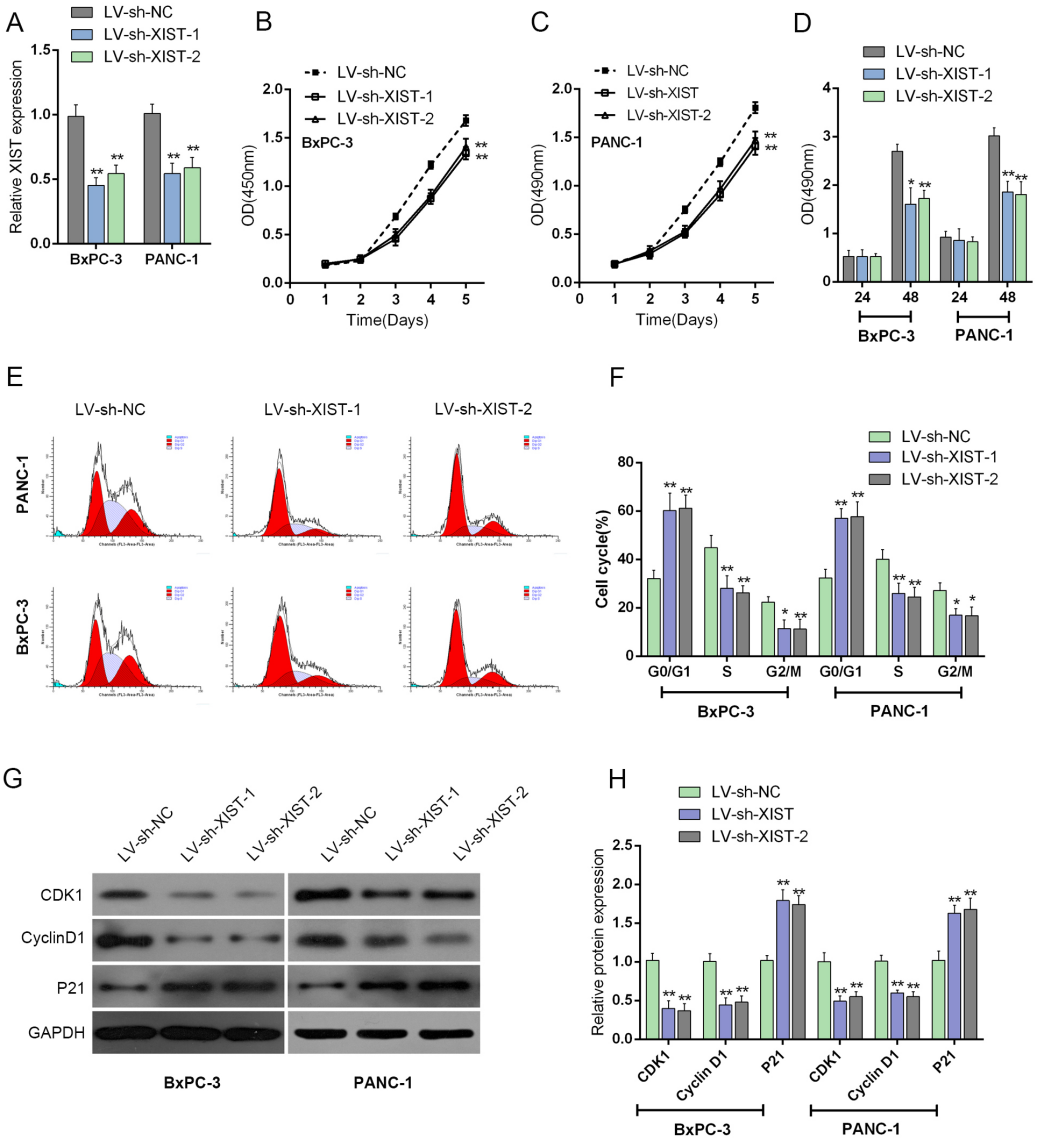
Knockdown of XIST induces cell cycle arrest at G0/G1 phase to suppress PC cell proliferation by regulating cell cycle-related genes in PC cell lines **(A)** LV-sh-XIST-1 and LV-sh-XIST-2 were transfected into BxPC-3 and PANC-1 cell to achieve XIST overexpression; the transfection efficiency was verified by using real-time PCR assays. **(B** and **C)** The cell viability of BxPC-3 and PANC-1 cells was determined by using MTT assays. **(D)** The cell proliferation of BxPC-3 and PANC-1 cells was determined by using BrdU assays. **(E)** Effect of XIST knockdown on cell cycle progression in BxPC-3 and PANC-1 cells was determined by using flow cytometric analysis. **(F)** Cell cycle distribution of BxPC-3 and PANC-1 cells in response to XIST knockdown. **(G** and **H)** The protein levels of iASPP, CDK1 and P21 in BxPC-3 and PANC-1 cells in response to XIST knockdown. The data are shown as mean± SD of three independent experiments. ^*^P<0.05, ^**^P<0.01.

To further investigate the exact role of XIST in PC, we achieved XIST silence by infecting LV-sh-XIST-1 and LV-sh-XIST-2 into BxPC-3 and PANC-1 cells. The expression of XIST was verified using real-time PCR assay. XIST expression was significantly down-regulated by both LV-sh-XIST-1 and LV-sh-XIST-2, compared with that of the LV-sh-NC (negative control) (Figure 2A). Then the cell viability and proliferation of BxPC-3 and PANC-1 cell lines were determined by using MTT and BrdU assays. Results showed that LV-sh-XIST-1- or LV-sh-XIST-2-induced XIST knockdown significantly suppressed the cell viability and proliferation of BxPC-3 and PANC-1 cell lines, compared with LV-sh-NC group (Figure 2B and 2C).

Flow cytometry was used to identify LV-sh-XIST-1 and LV-sh-XIST-2 transfection-induced changes in the cell cycle of PC cells. As shown in Figure 2D, the knockdown of XIST led to an arrest in G0/G1 phase. The percentages of cells in G0/G1 phase were increased up to about 60% (BxPC-3) and 58% (PANC-1) (Figure 2E and 2F), whereas those cells in G2/M phase decrease significantly (Figure 2E and 2F). Besides, the possible molecular mechanism for cell growth inhibition and cell cycle arrest in PC cells was also investigated. The protein levels of cell cycle regulatory genes, including iASPP, CKD1 and P21 were examined by using Western blot assays (Figure 2G and 2H). With its cyclin partners, CDK1 forms complexes that phosphorylate a variety of target substrates; phosphorylation of these proteins leads to cell cycle progression [16]. P21 is a cyclin-dependent kinase inhibitor that can complex with a variety of cyclins, resulting in cell cycle arrest. After LV-sh-XIST-1 or LV-sh-XIST-2-induced XIST knockdown, P21 protein level was significantly upregulated, while iASPP and CDK1 protein levels were downregulated (Figure 2G and 2H). The result demonstrated that knockdown of XIST induces cell cycle arrest at G0/G1 phase by regulating cell cycle-related genes, P21 and CDK1 in PC cell lines. Besides, LV-sh-XIST-1 and LV-sh-XIST-2 can both cause specific knockdown of XIST, thus we used LV-sh-XIST-1 as XIST shRNA in following experiments.

### Knockdown of XIST reduces tumor volume in the nude mouse xenograft model

**Figure 3:**
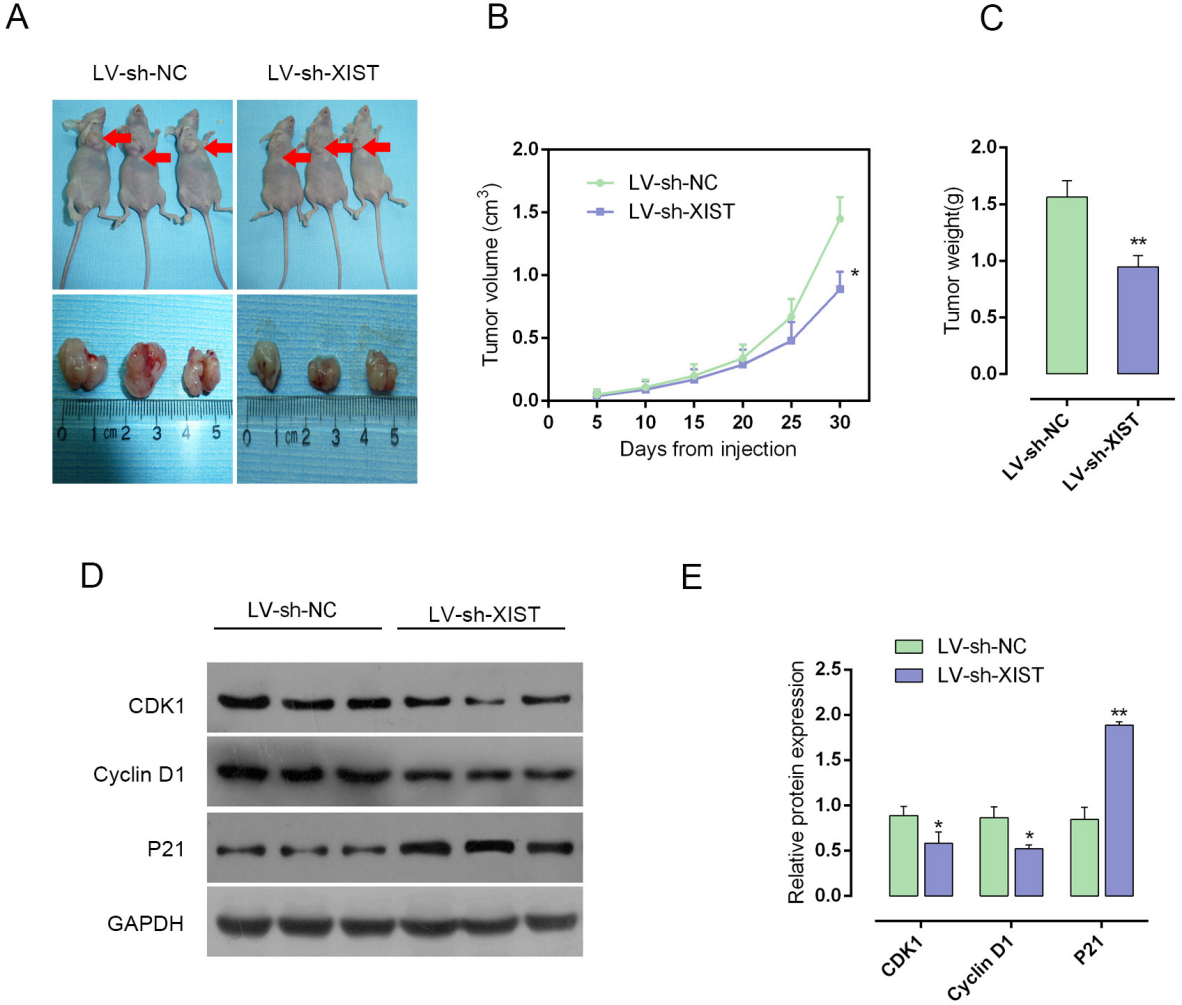
Knockdown of XIST reduces tumor volume in nude mouse xenograft model **(A)** Nude mouse xenograft model was generated. The lentivirus containing LV-sh-contr or LV-sh-XIST was injected into tumor after 5 days of PANC-1 cells injection, and the tumor volume changes on day 5, 10, 15, 20, 25 and 30 were determined in response to XIST knockdown. **(B)** Tumor volume in nude mice. **(C)** Tumor weight in nude mice. **(D** and **E)** The protein levels of CDK1, Cyclin D1 and P21 in xenograft tumors were determine by using Western blot assays. The data are shown as mean± SD of three independent experiments. ^*^P<0.05, ^**^P<0.01.

To further investigate the function of XIST in PC progression *in vivo*, the nude mouse xenograft model was generated. The lentivirus containing LV-sh-contr or LV-sh-XIST was injected into tumor after 5 days of PANC-1 cell injection, and the tumor volume and weight changes on day 5, 10, 15, 20, 25 and 30 were determined in response to XIST knockdown. After XIST knockdown, mice tumor size and weight was reduced, compared with LV-sh-contr infected mouse tumor (Figure 3A-3C). Besides, the protein levels of CDK1, Cyclin D1 and P21 in xenograft tumors were determined by using Western blot assays. Results showed that in LV-sh-XIST infected mouse tumor, CDK1 and Cyclin D1 protein levels were reduced, whereas P21 protein level was increased, compared with LV-sh-contr infected mouse tumor (Figure 3D and 3E). These data indicated that XIST knockdown suppressed PC tumor growth through inducing cell cycle arrest.

### iASPP is involved in the process of XIST regulating cell cycle-related proteins

**Figure 4:**
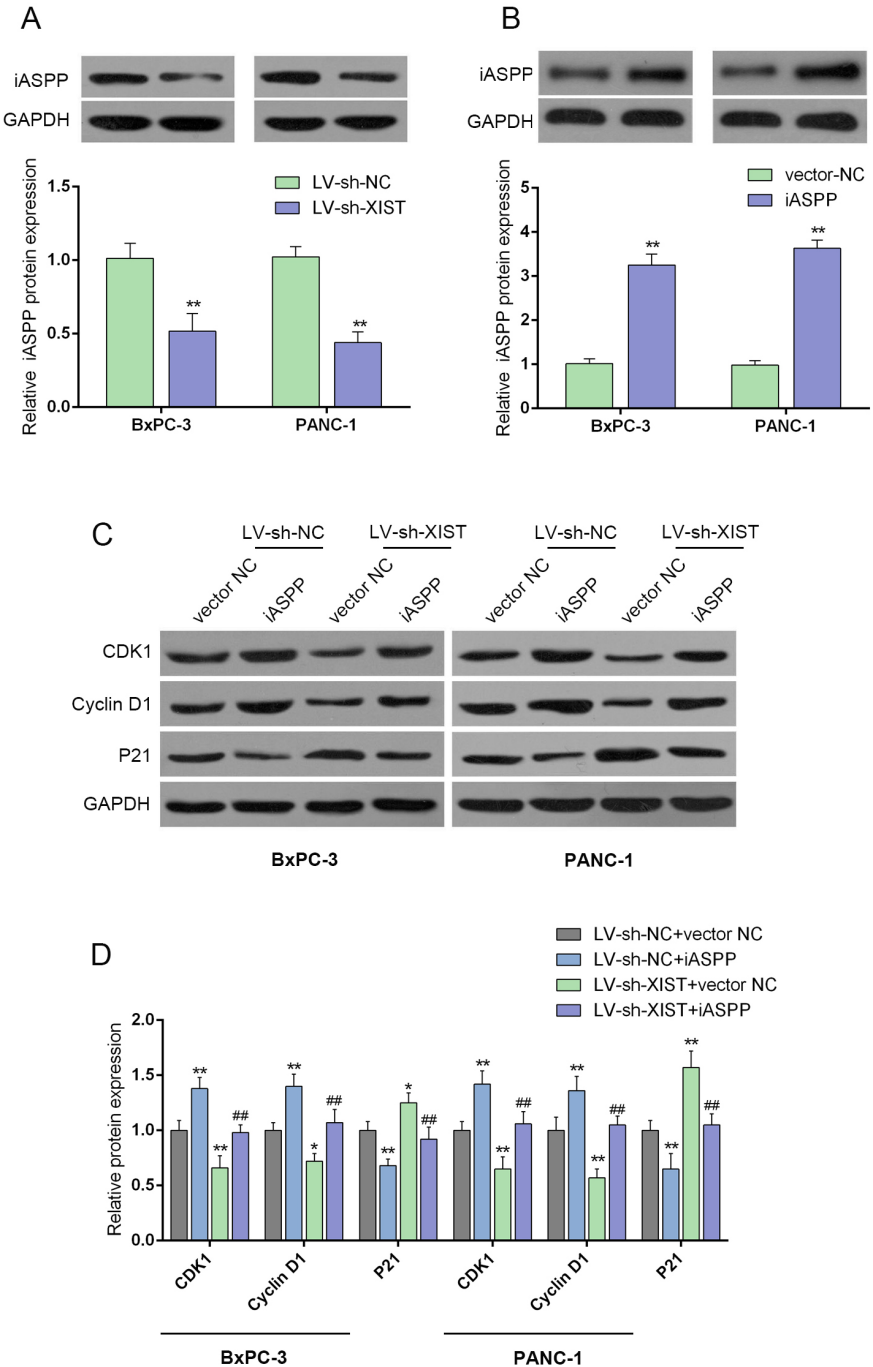
iASPP is involved in XIST regulating cell cycle-related proteins **(A** and **B)** The protein levels of iASPP in response to XIST knockdown in BxPC-3 and PANC-1 cells were determined by using Western blot assays. **(C** and **D)** The effect of co-processing iASPP vector and LV-sh-XIST on the protein levels of CDK1, Cyclin D1 and P21 in BxPC-3 and PANC-1 cells. The data are shown as mean± SD of three independent experiments. ^*^P<0.05, ^**^P<0.01, ^##^*P*<0.01.

iASPP interacts with the three members of the p53 family (p53, p63, and p73) [17], which was reported to play a central role in regulating cell cycle arrest, apoptosis, and DNA repair in a variety of cells [18]. To investigate the mechanism by which XIST regulate PC cell cycle, we validated whether iASPP was involved in the regulation. The protein levels of iASPP in LV-sh-XIST-infected BxPC-3 and PANC-1 cells, iASPP protein levels were significantly reduced, compared with LV-sh-contr group, as shown by Western blot assays (Figure 4A and 4B). The effect of co-processing iASPP vector and LV-sh-XIST on the protein levels of CDK1, Cyclin D1 and P21 in BxPC-3 and PANC-1 cells were then measured by using Western blot assays. Forced iASPP expression significantly upregulated CDK1 and Cyclin D1 protein levels, downregulated P21 protein level; the effect of XIST knockdown on the indicated proteins could be partially reversed by the forced iASPP expression (Figure 4C and 4D). These data suggested that iASPP is involved in the regulation of PC cell cycles.

### XIST inversely mutual-regulates miR-140/ miR-124

**Figure 5:**
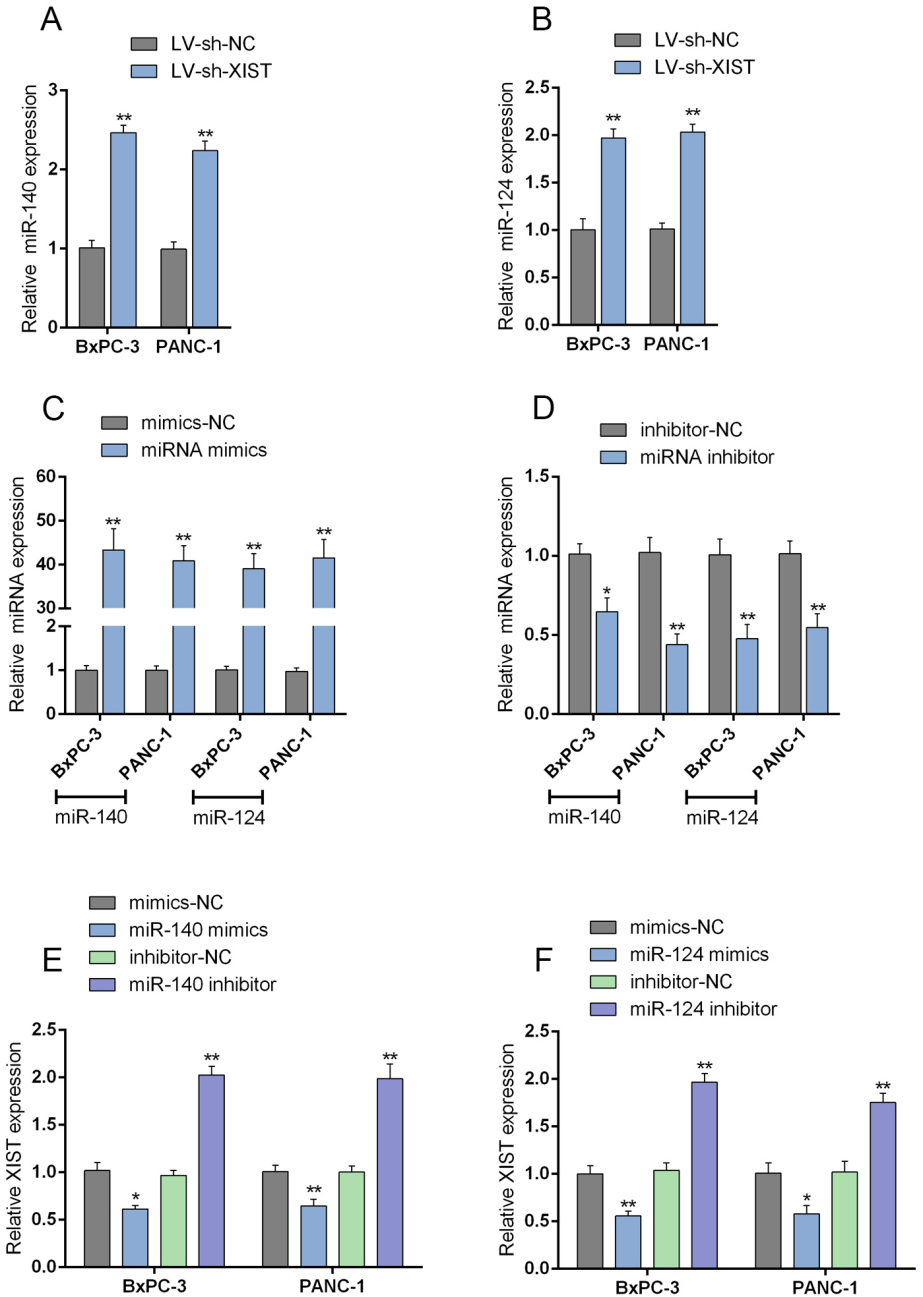
XIST inversely mutual-regulates miR-140/miR-124 **(A)** The expression of miR-140 in BxPC-3 and PANC-1 cells in response to XIST knockdown was determined by using real-time PCR assays. **(B)** The expression of miR-124 in BxPC-3 and PANC-1 cells in response to XIST knockdown was determined by using real-time PCR assays. **(C)** miR-140/miR-124 mimics were transfected into BxPC-3 and PANC-1 cells to achieve miR-140 and miR-124 overexpression, respectively. **(D)** miR-140/miR-124 inhibitor was transfected into BxPC-3 and PANC-1 cells to achieve miR-140 and miR-124 inhibition, respectively. **(E)** miR-140 mimics or miR-140 inhibitor was transfected into BxPC-3 and PANC-1 cells; XIST expression in BxPC-3 and PANC-1 cells in response to miR-140 overexpression or inhibition was determined by using real-time PCR assays. **(F)** miR-124 mimics or miR-124 inhibitor was transfected into BxPC-3 and PANC-1 cells; XIST expression in BxPC-3 and PANC-1 cells in response to miR-124 overexpression or inhibition was determined by using real-time PCR assays. The data are shown as mean± SD of three independent experiments. ^*^P<0.05, ^**^P<0.01.

Previous studies reported that XIST is associated with cancers through regulating miRNA [19]. In our previous study, we demonstrated that miR-140 inhibits cell growth and invasion in pancreatic duct adenocarcinoma by targeting iASPP [20]; in addition, downregulation of miR-124 predicts poor prognosis in pancreatic ductal adenocarcinoma patients [21]. To further investigate the mechanism by which XIST affected the cell cycle of PC cells, we validated whether XIST could regulate miR-140/miR-124 expression. In LV-sh-XIST-transfected BxPC-3 and PANC-1 cells, the expression levels of miR-140 and miR-124 were significantly upregulated (Figure 5A and 5B). MiR-140/miR-124 mimics or inhibitor was transfected into BxPC-3 and PANC-1 cells to achieve miR-140/miR-124 overexpression or inhibition, respectively; the transfection efficiency was verified by using real-time PCR assays (Figure 5C and 5D). Next, the XIST expression in miR-140/miR-124 mimics- or inhibitor-transfected BxPC-3 and PANC-1 cells was determined by using real-time PCR. Results showed that in BxPC-3 and PANC-1 cells ectopic miR-140/miR-124 expression significantly downregulated XIST expression, while miR-140/miR-124 inhibition upregulated XIST expression (Figure 5E and 5F). These data indicated that XIST inversely mutual-regulates miR-140/miR-124 expression in PC cell lines.

### MiR-140/miR-124 could directly bind to XIST and the 3’UTR of iASPP

**Figure 6:**
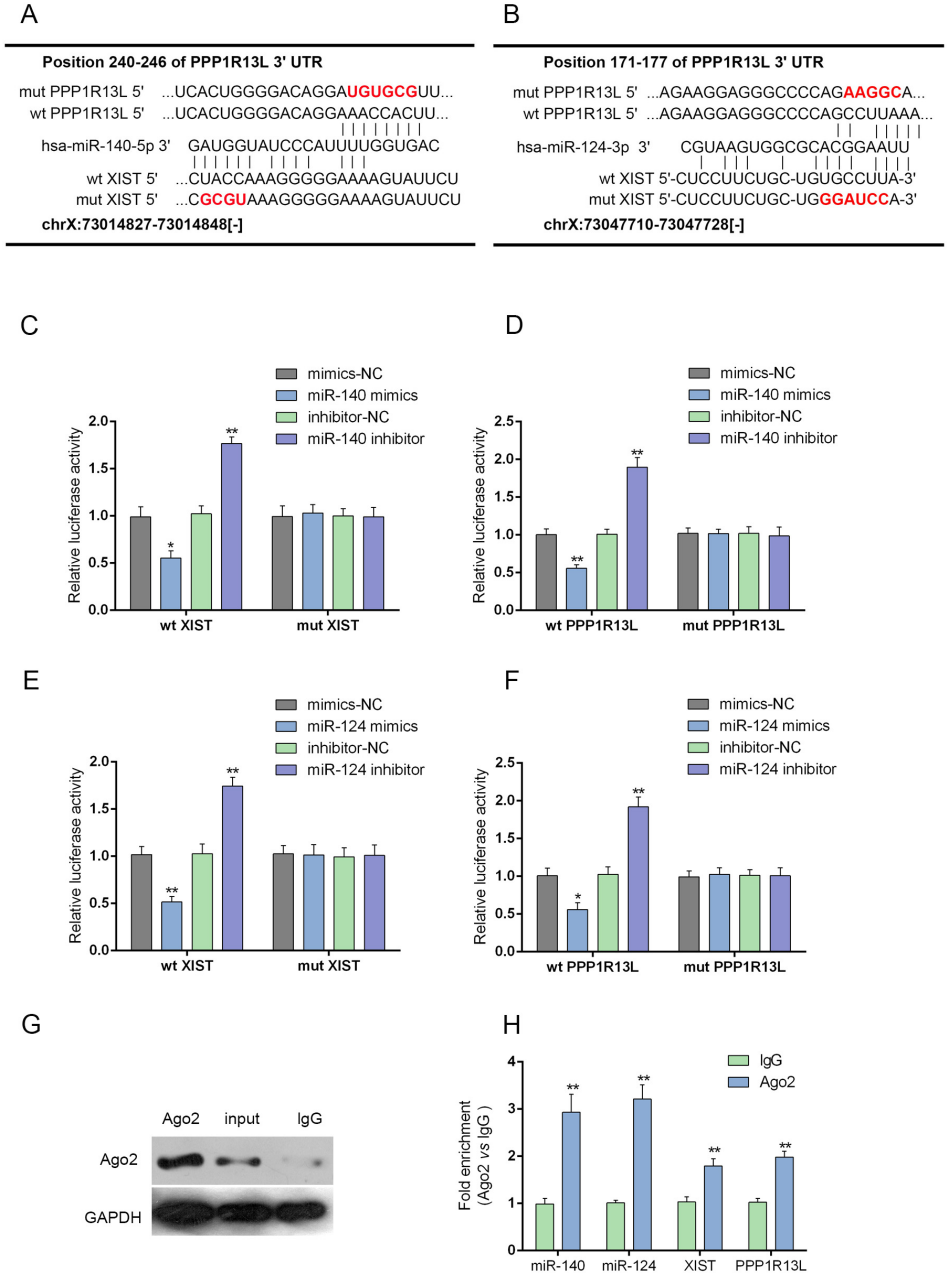
MiR-140/miR-124 could directly bind to XIST and the 3’UTR of iASPP **(A** and **B)** A wt-XIST luciferase reporter gene vector, a mut-XIST vector containing a 4 bp mutation in the predicted binding site of miR-140, or a 6 bp mutation in the predicted binding site of miR-124, a wt-iASPP 3’UTR luciferase reporter gene vector, as well as a mut-iASPP 3’UTR vector containing a 6 bp mutation in the predicted binding site of miR-140, or a 5 bp mutation in the predicted binding site of miR-124 was constructed. **(C** and **D)** The indicated luciferase reporter gene vectors were co-transfected into PANC-1 cells with miR-140 mimics or inhibitor; the luciferase activity was determined by using dual luciferase assays. **(E** and **F)** The indicated luciferase reporter gene vectors were co-transfected into PANC-1 cells with miR-124 mimics or inhibitor; the luciferase activity was determined by using dual luciferase assays. **(G** and **H)** Association of XIST, miR-140/miR-124 and iASPP with AGO2. PANC-1 cellular lysates were used for RNA immunoprecipitation with AGO2 antibody. Detection of AGO2 and IgG using Western blot (up); detection of XIST, miR-140/miR-124 and iASPP using qRT-PCR (low). All data of XIST and iASPP expression were normalized to β-actin mRNA expression levels. MiR-140/miR-124 expression data was normalized to U6 small RNA expression. The data are shown as mean± SD of three independent experiments. ^*^P<0.05, ^**^P<0.01.

We demonstrated that miR-140 binds to the 3’UTR of iASPP to attenuate PC cell growth and invasion capacity in our previous study [20]. In addition, miR-124 regulates the proliferation of colorectal cancer cells by targeting iASPP [22]. Here, we generated luciferase assays to investigate whether miR-140/miR-124 binds to XIST and iASPP to regulate their expression. A wt-XIST luciferase reporter gene vector, a mut-XIST vector containing a 4 bp mutation in the predicted binding site of miR-140, or a 6 bp mutation in the predicted binding site of miR-124, a wt-iASPP 3’UTR luciferase reporter gene vector, as well as a mut-iASPP 3’UTR vector containing a 6 bp mutation in the predicted binding site of miR-140, or a 5 bp mutation in the predicted binding site of miR-124 was constructed (Figure 6A and 6B). The indicated luciferase reporter gene vectors were co-transfected into PANC-1 cells with miR-140 mimics/miR-140 inhibitor (Figure 6C and 6D) or miR-124 mimics/miR-124 inhibitor (Figure 6E and 6F). Then the luciferase activity was determined by using dual luciferase assays. Results showed that the luciferase activity of wt-XIST and wt-iASPP 3’UTR vectors were significantly suppressed by miR-140/miR-124 mimics, increased by miR-140/miR-124 inhibitors; the changes of luciferase activity were abolished by mutations in miR-140 or miR-124 binding sites in XIST or the 3’UTR of iASPP (Figure 6E and 6F). Further, the interaction between XIST and miR-140/miR-124, between miR-140/miR-124 and iASPP was validated using RNA immunoprecipitation assays with the AGO2 antibody. As exhibited by Western blot assays, AGO2 protein could be precipitated from the cellular extract (Figure 6G). In RNA extracted from the precipitated AGO2 protein, we could detect XIST, miR-140/miR-124 and iASPP with a 1.8∼3-folds enrichment compared to IgG (Figure 6H), indicating that miR-140/miR-124 could directly bind to XIST and the 3’UTR of iASPP.

### XIST regulates cell cycle-related genes through miR-140/miR-124

**Figure 7:**
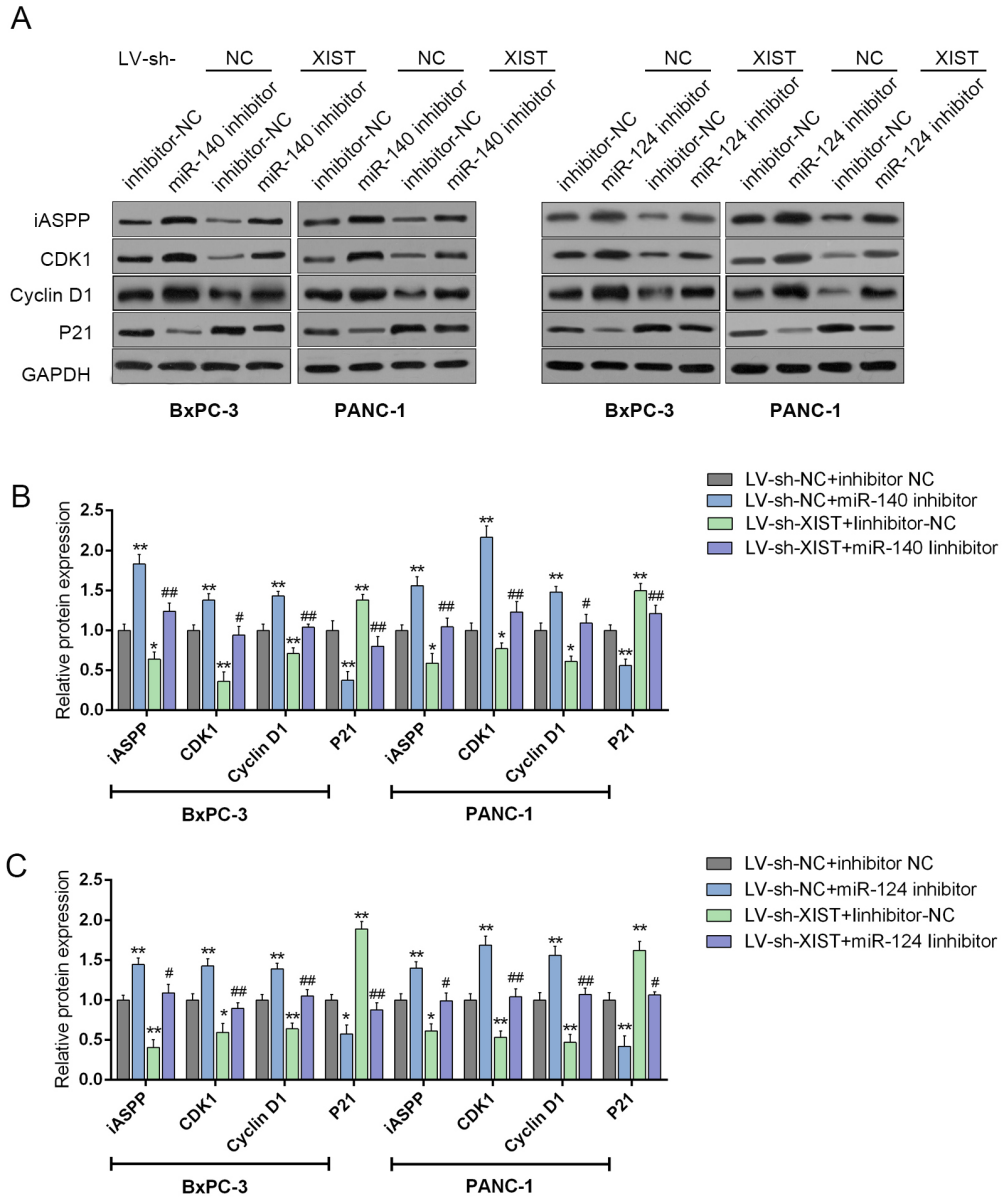
XIST regulates cell cycle-related genes through miR-140/miR-124 **(A)** si-iASPP was transfected into BxPC-3 and PANC-1 cells to achieve iASPP knockdown; the protein levels of iASPP, CDK1, P21 and P27 in BxPC-3 and PANC-1 cells were determined by using Western blot assays. **(B** and **C)** and LV-sh-XIST and miR-140 inhibitor or miR-124 inhibitor were co-transfected into BxPC-3 and PANC-1 cells, respectively; the protein levels of iASPP, CDK1, P21 and P27 in BxPC-3 and PANC-1 cells were determined by using Western blot assays. The data are shown as mean± SD of three independent experiments. ^*^P<0.05, ^**^P<0.01, ^#^*P*<0.05, ^##^*P*<0.01.

We have demonstrated that knockdown of XIST induces cell cycle arrest at G0/G1 phase by regulating cell cycle-related genes in PC cell lines; given that miR-140/miR-124 could directly bind to XIST and the 3’UTR of iASPP, we then investigated whether XIST regulates cell cycle-related genes through miR-140/miR-124/iASPP. BxPC-3 and PANC-1 cells were co-transfected with LV-sh-XIST and miR-140 inhibitor or miR-124 inhibitor; then the protein levels of iASPP and cell cycle-related factors, P21, CDK1 and Cyclin D1 were determined by using Western blot assays (Figure 7A-7C). Results showed that miR-140 inhibition or miR-124 inhibition could upregulate the protein levels of iASPP, Cyclin D1 and CDK1, downregulated the protein levels of P21; XIST knockdown could downregulate the protein levels of iASPP, Cyclin D1 and CDK1, upregulate the protein levels of P21; the effects of miR-140 or miR-124 inhibitor on the indicated protein levels could be partially reversed by XIST knockdown (Figure 7A-7C). These data suggested that XIST might regulate cell proliferation- and cell cycle-related genes through miR-140/miR-124.

### iASPP inhibits the transcriptional activity of p73 to promote XIST expression

**Figure 8:**
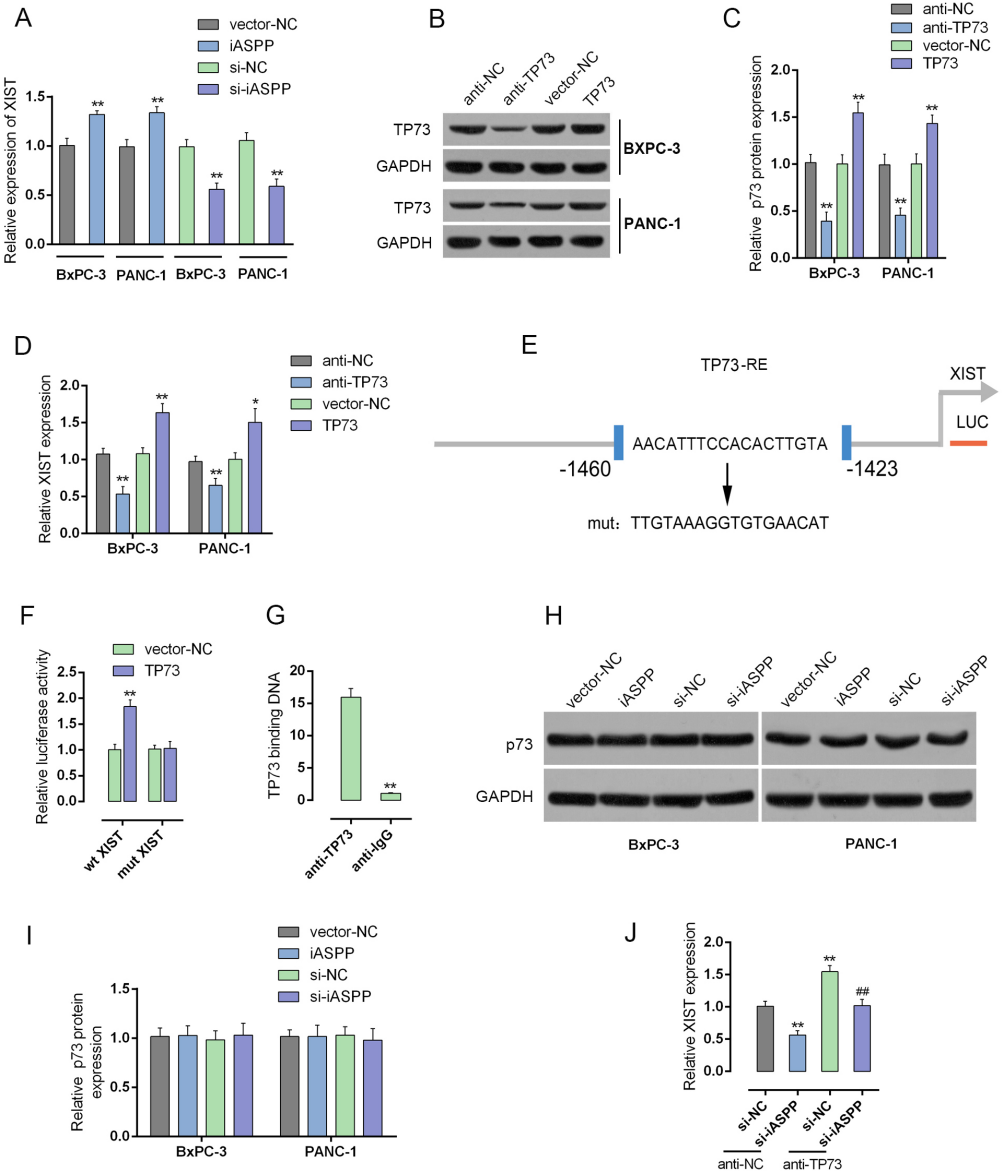
iASPP inhibits the transcriptional activity of p73 to promote XIST expression **(A)** forced iASPP or iASPP knockdown was achieved by introducing iASPP vector or si-iASPP into BxPC-3 and PANC-1 cells. The expression levels of XIST were then determined by using real-time PCR assays. **(B** and **C)** p73 knockdown or p73 overexpression was achieved by transfection of anti-TP73 or TP73 vector, as verified using real-time PCR and Western blot assays. **(D)** XIST expression in response to p73 knockdown or p73 overexpression was determined by using real-time PCR assays. **(E)** A wt-XIST promoter and a mut-XIST promoted luciferase reporter gene vector were constructed by mutating the putative binding site of p73 in XIST promoter. **(F)** The indicated vectors were co-transfected with pcDNA3.1/p73 into PANC-1 cells. The luciferase activity was determined by using dual luciferase assays. **(G)** The real-time ChIP assay showed that the level of p73 antibody binding to XIST promoter was much greater than that of IgG. **(H** and **I)** forced iASPP or iASPP knockdown was achieved by introducing iASPP vector or si-iASPP into BxPC-3 and PANC-1 cells. The protein levels of p73 were determined by using Western blot assays. **(J)** XIST expression in response to co-processing si-iASPP and anti-*TP73* was determined by using real-time PCR assays. ^**^P<0.01, ^##^*P*<0.01.

Given the involvement of iASPP in XIST regulating PC cell cycle arrest, we further investigated the mechanism by which XIST and iASPP exerted the joint function. In BxPC-3 and PANC-1 cells, the expression levels of XIST in response to forced iASPP expression and iASPP knockdown were determined by using real-time PCR assays. XIST expression was upregulated by forced iASPP, while downregulated by iASPP knockdown, indicating that iASPP could positively regulate the XIST expression (Figure 8A).

iASPP is an apoptotic-specific regulator of p53. Typically, iASPP suppresses apoptosis by inhibiting the transactivation function of p53 on the promoters of pro-apoptotic genes [17]. Recently, it has been reported that p73 structural resemblance to p53 and was the first identified homologue of the tumor suppressor gene p53 [23, 24]. In addition, iASPP inhibits apoptosis independently of p53 in tumor cells, mainly by inhibiting the transcriptional activity of p63/p73 [17]. Here, we performed a series of mechanistic assays to investigate whether p73 plays a role in the joint function of XIST and iASPP. Knockdown or overexpression of p73 was achieved through introducing anti-*TP73* or *TP73* vector into BxPC-3 and PANC-1 cells, as verified using real-time PCR and Western blot assays (Figure 8B and 8C); the expression levels of XIST in the two cell lines were then monitored. XIST expression was significantly upregulated by anti-*TP73*-induced p73 knockdown, whereas downregulated by *TP73* vector-induced p73 overexpression, compared with anti-NC (Figure 8D), suggesting that p73 might inversely regulate XIST. To confirm this hypothesis, we constructed a wt-XIST promoter luciferase reporter gene vector, as well as a mut-XIST promoter vector containing a mutant segment within the promoter of XIST (Figure 8E). The indicated vectors were co-transfected into PANC-1 cells with pcDNA3.1/p73, and then the luciferase activity was determined by using dual luciferase assays. Results showed that the luciferase activity of wt-XIST promoter vectors was significantly reduced by p73; after the mutation within the promoted of XIST, p73-induced suppression of luciferase activity was abolished (Figure 8F). Furthermore, the real-time ChIP assay showed that the level of p73 antibody binding to the binding element in the XIST promoter was much greater than that of IgG (Figure 8G), indicating that p73 binds to the promoter of XIST to inhibit its expression. We also investigated whether iASPP could inhibit the transcriptional activity of p73. The protein levels of p73 in response to forced iASPP expression and knockdown were determined by using Western blot assays. The protein levels of p73 showed no significant changes, either in response to forced iASPP expression or iASPP knockdown (Figure 8H and 8I). However, after co-transfection of anti-*TP73* and si-iASPP into BxPC-3 and PANC-1 cells, XIST expression was significantly altered. XIST expression was reduced by si-iASPP transfection, increased by *TP73*; the suppressive effect of si-iASPP on XIST could be partially reversed by *TP73* (Figure 8J), indicating that iASPP actually enhanced the transcriptional activity of p73, without protein level change, to enhance the promotive effect of p73 on XIST transcriptional activity.

### The expression levels of miR-140, miR-124, iASPP mRNA, CDK1 mRNA and p21 mRNA in PC tissues and their correlations with XIST

**Figure 9:**
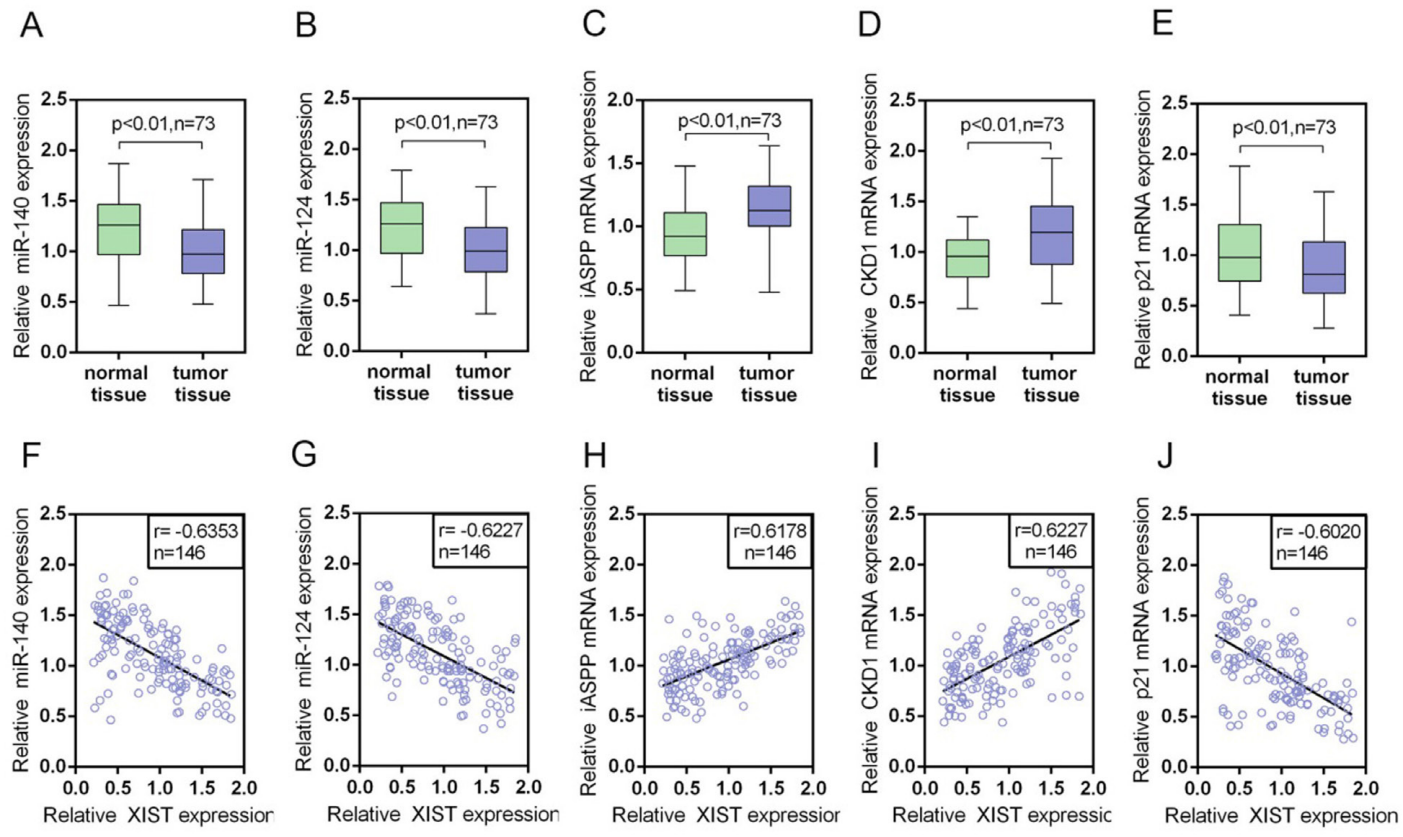
The expression levels of miR-140, miR-124, iASPP mRNA, CDK1 mRNA and p21 mRNA in PC tissues and their correlations with XIST **(A**-**E)** The expression levels of miR-140, miR-124, iASPP mRNA, CDK1 mRNA and P21 mRNA in a large panel of 73 paired PC tissues and matched adjacent normal tissues were determined by using real-time PCR assays. The data are shown as mean± SD of three independent experiments. **(F**-**J)** By performing Spearman’s rank correlation analysis, the correlation between XIST and miR-140, XIST and miR-124, XIST and iASPP, XIST and CDK1, XIST and P21 was analyzed.

We determined the expression levels of miR-140, miR-124, iASPP mRNA, CDK1 mRNA and p21 mRNA in PC tissues and adjacent normal tissues by using real-time PCR assays. Results showed that miR-140, miR-124 and p21 mRNA expression was downregulated, while iASPP and CDK1 mRNA expression was upregulated in PC tissues compared with normal tissues (Figure 9A-9E). By using Spearman’s rank correlation analysis, we observed that XIST was inversely correlated with miR-140, miR-124 and p21, respectively, positively correlated with iASPP and CDK1, respectively (Figure 9E-9I).

## DISCUSSION

In recent years, accumulating evidence has demonstrated that XIST is aberrantly expressed in a variety of malignant solid tumors [7]. Clinicopathological analysis has shown that over-expression of XIST correlates with tumor progression [25–27]. For instance, high XIST predict poor outcome after high-dose alkylating chemotherapy in patients with a BRCA1-like breast cancer [27]. XIST acts as an oncogene in non-small cell lung cancer by epigenetically repressing KLF2 expression [28]. In the present study, we demonstrated a significant higher expression level of XIST in the PC tissues and cell lines, compared to the normal tissues and cell line. High XIST expression was related to poorer clinicopathologic features and shorter OS and DFS. In addition, after successfully silencing XIST by LV-sh-XIST transfection, the viability and proliferation of PC cells was significantly suppressed in response to XIST silence, suggesting the key role of XIST in maintaining PC cell proliferation. In order to investigate the mechanism by which XIST affects PC cell proliferation, we revealed that XIST knockdown led to an arrest in G0/G1 phase. The percentages of cells in G0/G1 phase were significantly increased, whereas those cells in G2/M phase decrease significantly. Then we investigated whether XIST affected PC cell cycle arrest through cell cycle-related genes. After XIST knockdown, CDK1 and Cyclin D1 protein levels were downregulated, whereas P21 protein level was upregulated, indicating that XIST may regulate cell cycle-related CDK1, Cyclin D1 and P21 to modulate PC cell cycle arrest. In addition, results from *in vivo* nude mouse xenograft tumor model showed that XIST knockdown reduced the tumor size, the protein levels of CDK1 and Cyclin D1 in tumors, and increased P21 protein level in tumors. These data all indicated the key role of XIST in regulating PC cell cycle so as to affect PC cell growth and cancer progression.

Tumor suppressor p53 plays a central role in regulating cell cycle arrest, apoptosis, and DNA repair in a variety of cells [18]. The importance of p53 in tumor suppression is highlighted by the observation that about half of all human cancers show evidence for the loss of normal p53 function due to mutation within the p53 gene. Most p53 mutations map to the DNA-binding domain, therefore, they are unable to activate genes up-regulated by wild-type p53, a loss of function that ultimately leads to unchecked cell division [29]. iASPP is overexpressed in acute leukemia’s regardless of p53 mutation status [30], suggesting that iASPP may promote carcinogenesis by other mechanisms additional to p53 inhibition. Thus, it is very important to understand the function of iASPP as an oncoprotein in a p53-independent manner. However, to date, most attention has been focused on how iASPP interacts with the three members of the p53 family (p53, p63, and p73), and the biological significance of its interactions with other proteins remains largely to be determined. Given the importance of iASPP of interacting with the three members of the p53 family (p53, p63, and p73), and the major role of p53 in cell cycle arrest, we further investigated whether iASPP was involved in the regulation of PC cell cycles. In LV-sh-XIST-infected BxPC-3 and PANC-1 cells, iASPP protein levels were significantly reduced by XIST knockdown; forced iASPP expression could reduce CDK1 and Cyclin D1 protein levels, increase P21 protein level, as well as partially reverse the effect of XIST knockdown on cell cycle-related proteins. These all suggested the involvement of iASPP in XIST regulating PC cell cycle.

Alterations of miRNAs expression are implicated in almost all fields of cancer biology, including cell growth, apoptosis, migration and/or invasion, and they can function as either tumor suppressors or oncogenes [31]. The impact of specific miRNAs on cancer biology depends on their downstream targets [32, 33]. Moreover, emerging evidences have revealed that the mutual regulation between miRNAs and lncRNAs play major roles in tumor progression [34]. To investigate the mechanism by which XIST regulate iASPP and cell cycle-related gene expression, we focused on two PC cell proliferation-related miRNAs: miR-140 and miR-124 [20, 21]. In PC cell lines, XIST and miR-140/miR-124 could inversely regulate each other, respectively. Moreover, miR-140 and miR-124 could directly bind to XIST and the 3’UTR of iASPP, respectively. To validate whether miR-140 and miR-124 were involved in XIST regulating PC cell cycle arrest through iASPP, we determined the protein levels of iASPP, CDK1, Cyclin D1 and P21. Si-iASPP-induced iASPP knockdown downregulated iASPP, CDK1 and Cyclin D1 protein levels while upregulated P21 protein level. We demonstrated that miR-140 inhibits PC cell proliferation through direct binding to iASPP [20]; consistent with our previous study, we observed that miR-140 inhibition upregulated iASPP, CDK1 and Cyclin D1 protein levels while downregulated P21 protein level. According to previous studies, silencing of the miR-124 genes facilitates pancreatic cancer progression and metastasis [35]. In the present study, we observed similar results: miR-124 inhibition upregulated iASPP, CDK1 and Cyclin D1 protein levels while downregulated P21 protein level. Moreover, the effects of LV-sh-XIST on the indicated protein levels could be partially reversed by miR-140 or miR-124 inhibitor, respectively. These data suggested that miR-140 and miR-124 were involved in the process of XIST regulating iASPP and cell cycle-related genes.

iASPP is an apoptotic-specific regulator of p53. Typically, iASPP suppresses apoptosis by inhibiting the transactivation function of p53 on the promoters of pro-apoptotic genes [17]. Recently, it has been reported that p73 structural resemblance to p53 and was the first identified homologue of the tumor suppressor gene p53 [23, 24]. In addition, iASPP inhibits apoptosis independently of p53 in tumor cells, mainly by inhibiting the transcriptional activity of p73 [17]. In the present study, iASPP positively regulated XIST expression in BxPC-3 and PANC-1 cells; then we investigated whether p73 was involved in this process. After p73 knockdown by anti-*TP73*, XIST expression was upregulated, suggesting p73 might inhibit XIST expression. As confirmed by luciferase and real-time ChIP assays, p73 could bind to the promoter of XIST to inhibit XIST expression. Moreover, we revealed that iASPP suppressed the transcriptional activity of p73 to suppress the inhibitory effect of p73 on XIST expression, but without changing the protein level of p73. The *TP73* and *TP63* genes are expressed in different N-terminal isoforms either with proapoptotic (transactivation domain, TA) and antiapoptotic (N-terminally truncated, ΔN) function [36]; these isoforms express at different levels under different contexts. According to previous studies, iASPP and p63 (both ΔN and TA) are linked in an autoregulatory feedback loop [37]. Here, we hypothesize that iASPP might regulate the expression of different p73 isoforms, so as to affect the role of p73 shown without the total p73 protein changes. However, this hypothesis needs further investigation.

**Figure 10:**
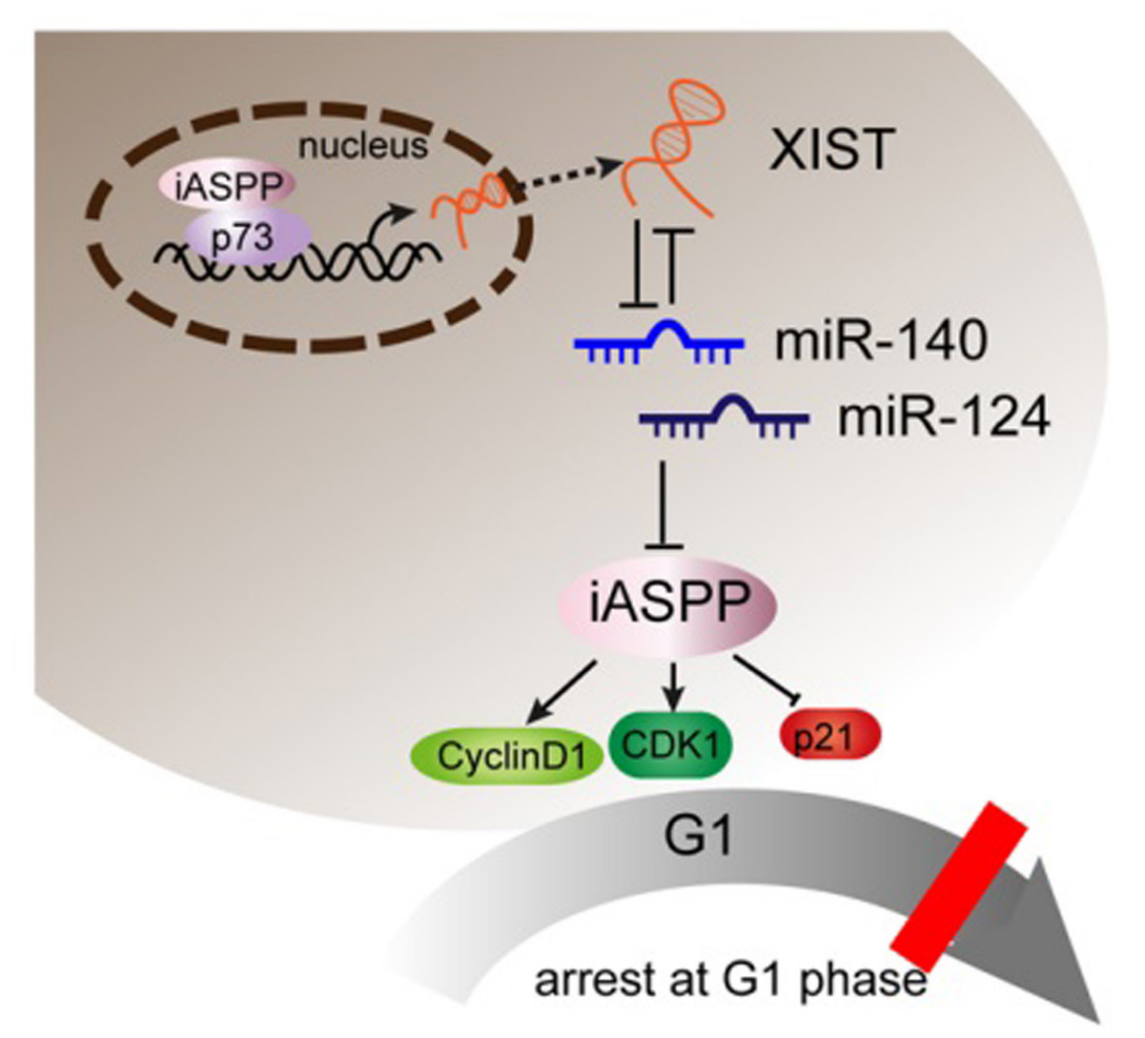
A schematic diagram showing XIST inhibits miR-140/miR-124 to promote pancreatic carcinoma growth through iASPP and cell cycle-related factors

We also determined the expression levels of miR-140, miR-124, iASPP, CDK1 and P21 in PC tissues and adjacent normal tissues. In PC tissues, miR-140, miR-124 and P21 expression was downregulated, while iASPP and CDK1 expression was upregulated. XIST was positively related to iASPP and CDK1, inversely related to miR-140, miR-124 and P21, respectively. In summary, it was identified that XIST expressed at a higher level in PC than the normal tissues and cell lines. XIST knockdown inhibits PC cell proliferation an tumor size through leading to an arrest in G0/G1 phase through regulating cell cycle arrest-related CDK1 and P21, and p53-independent apoptosis-related factor iASPP. miR-140 and miR-124 were involved in this process through direct binding to XIST and the 3’UTR of iASPP. Besides, the first identified homologue of the tumor suppressor gene p53, p73, binds to the promoter of XIST to inhibit XIST expression; iASPP suppresses p73 transcriptional activity to suppress the inhibitory effect of p73 on XIST without changing p73 protein levels. (See mechanism diagram shown in Figure 10) Taken together, XIST plays a key role in regulating PC cells proliferation and cell cycle, and may provide a potential therapeutic strategy for PC.

## MATERIALS AND METHODS

### Tissue samples, cell lines and cell infection/transfection

All sequences used in the present study are listed in the Supplementary Material (See Supplementary Table 1 Sequences used in the present study).

We collected 73 paired primary PC tissues and the matched adjacent normal tissues. We obtained all samples from patients who underwent surgical resection at Xiangya Hospital of Central South University (Changsha, China). The tissues were snap-frozen in liquid nitrogen, and then stored at -80°C. This project was approved by the Ethic Committee of Xiangya Hospital of Central South University.

We purchased pancreatic epithelial cell (CS-PE), human PC cell lines, including AsPC-1, BxPC3, SW1990, PANC-1, CAPAN-1 and CAPAN-2 cells from the American Type Culture Collection (ATCC, USA). Cells were cultured in RPMI-1640 medium (Invitrogen, USA) supplemented with 10% fetal bovine serum (Gibco, USA) at 37°C in a humidified atmosphere with 5% CO_2_.

Knockdown of XIST was achieved by infection of LV-sh-XIST (Genepharma, China); knockdown of iASPP was achieved by transfection of si-iASPP (Genepharma, China); forced iASPP expression was achieved by transfection of iASPP vector (Genepharma, China); p73 knockdown was achieve by anti-TP73 (Genepharma, China); forced p73 expression was achieved by pcDNA3.1/p73 (Genepharma, China); miR-140/miR-124 overexpression or inhibition was achieved by transfection of miR-140/miR-124 mimics or inhibitor (Genepharma, China) using Lipofectamine 2000 (Invitrogen, USA). Cells were plated in 6-well plates or 96-well plates, transfected, incubated for 48 h and used for further assays or RNA/protein extraction.

### Lentivirus production and infection

To generate XIST inhibited or control lentivirus, the pLVX vector containing shRNA XIST or the control sequence were co-transfected into 293 cells together with the plasmids pHelper1.0 and pHelper2.0 (Genechem) that contain the necessary elements for virus packaging. The transfection was generated using Lipofectamine 2000 (Invitrogen) following the manufacturer’s instructions. Supernatants containing the lentiviruses were harvested at 72 h, then concentrated with Lenti-Pac™ Lentivirus Concentration Kit (GeneCopoeia), the viral titers of the lentivirus were determined before infection. PC cells were plated at a concentration of 2 ×10^6^ cells/ml, incubated for 16 h. For infection, 1.5ml/well viral supernatant was used to replace the medium. Cells were incubated at 37 °C for 10 h, and then the fresh media was used to replace the viral supernatant. 48 h after the infection, 2mg/ml puromycin was used to select cells. 5 days later, the infection efficiency was verified using Western blot or real-time PCR assays. All sequences used are provided in Supplementary Table 1 (See Supplementary Table 1 Sequences used in the present study).

### Mouse xenograft model

All animal experiments were performed in accordance with a protocol approved by Xiangya Hospital of Central South University. Nude mice (4 weeks old) were provided by the laboratory animal center of Xiangya Hospital, Central South University. PC cells were suspended in PBS at 1×10^7^ cells/ml. 200μl of cell suspension were injected subcutaneously into the right axilla of nude mice. 7 days later, either lentivirus with shRNA XIST or lentivirus control (5×10^7^ particles/10 μl) was injected into tumors. Tumors were monitored by Vernier calipers on the indicated days. The tumor volume was calculated according to the formula: tumor volume (mm^3^) = length (mm) × width (mm) × height (mm) [16], and the tumors were paraffin-embedded. 30 days later, all mice were sacrificed and tumors were excised. Tumor tissue was collected for RNA extraction or immunohistochemical analysis.

### RNA extraction and SYBR green quantitative PCR analysis

We extracted total RNA from cells using Trizol reagent (Invitrogen, USA). The expression of XIST, iASPP mRNA and CDK1 mRNA in PC cell lines was measured by SYBR green qPCR assay (Takara, China). Data were processed using 2^-ΔΔCT^ method. Mature miR-140 and miR-124 expressions in cells was measured using a Hairpin-it TM miRNAs qPCR kit (Genepharma, Shanghai, China). We used expression of RNU6B as an endogenous control. Data were processed using 2^-ΔΔCT^ method. All primers used are provided in Supplementary Table 1 (See Supplementary Table 1 Sequences used in the present study).

### MTT assay

A modified MTT assay was used to evaluate cell viability. After seeding 2×10^3^ transfected cells/well into 96-well culture plates we assessed the viability of BxPC3 and PANC-1 cells transfected with LV-sh-contr or LV-sh-XIST at five time points (on day 1, 2, 3, 4 and 5). In brief, quantification of mitochondrial dehydrogenase activity was achieved through the enzymatic conversion of MTT [3-(4,5-dimethyldiazol-2-yl)-2,5- diphenyltetrazolium bromide; Sigma-Aldrich, MO, USA] to a colored formazan product. MTT (10 μl, 10 mg/ml) was added to the cells, incubated for 4 h, and we terminated the reaction by removal of the supernatant and addition of 100 μl DMSO to dissolve the formazan product. After 0.5 h, the optical density (OD) of each well was measured at 450 nm using a plate reader (ELx808 Bio-Tek Instruments, City, ST, USA).

### BrdU incorporation assay

DNA synthesis in proliferating cells was determined by measuring 5-Bromo-2-deoxyUridine (BrdU) incorporation. BrdU assays were performed at 24 h and 48 h after transfecting BxPC3 and PANC-1 cells with LV-sh-contr or LV-sh-XIST. After seeding the infected cells in 96-well culture plates at a density of 2 × 10^3^ cells/well, they were cultured for 24 h or 48 h, and incubated with a final concentration of 10 μM BrdU (BD Pharmingen, San Diego, CA, USA) for 2 h to 24 h. When the incubation period ended, we removed the medium, fixed the cells for 30 min at RT, incubated them with peroxidase-coupled anti-BrdU-antibody (Sigma-Aldrich) for 60 min at RT, washed them three times with PBS, incubated the cells with peroxidase substrate (tetramethylbenzidine) for 30 min, and measured the absorbance values at 490 nm. Background BrdU immunofluorescence was determined in cells not exposed to BrdU but stained with the BrdU antibody.

### Cell cycle analysis

Cell cycle distribution was determined by flow cytometry [38]. In brief, the uninfected and infected cells were harvested by trypsinization, washed with PBS and fixed in 70% ethanol at 4°C. Cells were collected by centrifugation and re-suspended in PBS containing 100 μg/ml RNase A and 40μg/ml PI, and then incubated at 4°C for 30 min in the dark. Cells were analyzed by flow cytometry using a FACSCalibur flow cytometer (Becton-Dickinson, San Jose, CA, USA). The fractions of the cells in G0/G1, S, and G2/M phases were analyzed using dedicated software (Becton–Dickinson, San Jose, CA, USA).

### Western blot analysis

The expression of iASPP, CDK1, P21 and P73 in PC cells was detected by performing Immunoblotting. We lysed cultured or transfected cells in RIPA buffer with 1% PMSF and loaded protein onto a SDS-PAGE minigel and transferred them onto PVDF membrane. After probed with the following antibodies: iASPP (ab34898, Abcam, MA, USA), CDK1 (Cat# A17, Abcam), P21 (Cat# EPR362, Abcam) and P73 (Cat# EP436Y, Abcam) at 4°C overnight, the blots were subsequently incubated with HRP-conjugated secondary antibody (1:5000). ECL Substrates was used to visualize signals (Millipore, MA, USA). β-actin was used as an endogenous protein for normalization.

### Chromatin immunoprecipitation (ChIP)

Briefly, the treated cells were cross-linked with 1% formaldehyde, sheared to an average size of 400 bp DNA, and immunoprecipitated using antibodies against p73 (Sigma-Aldrich). The ChIP-PCR primers were designed to amplify the promoter regions containing putative p73 binding sites within XIST promoter. A positive control antibody (RNA polymerase II) and a negative control non-immune IgG were used to demonstrate the efficacy of the kit reagents (Epigentek Group Inc., NY, USA, P-2025-48). The immunoprecipitated DNA was subsequently cleaned, released, and eluted. The eluted DNA was used for downstream applications, such as ChIP-PCR. The fold-enrichment (FE) was calculated as the ratio of the amplification efficiency of the ChIP sample to that of the non-immune IgG. The amplification efficiency of RNA Polymerase II was used as a positive control. FE% = 2 (IgG CT-Sample CT) × 100%.

### Luciferase reporter assay

PANC-1 cells were seeded into a 24-well plate. After cultured overnight, cells were co-transfected with the wild-type and mutated XIST or wild-type and mutated *PPP1R13L* 3’UTR reporter plasmid or pRL-TK plasmids and miR-140/miR-124 mimics or miR-140/miR-124 inhibitor. Luciferase assays were performed 48 h after transfection using the Dual Luciferase Reporter Assay System (Promega, WI, USA). All sequences are provided in Supplementary Table 1 (See Supplementary Table 1 Sequences used in the present study).

### RNA immunoprecipitation

RNA immunoprecipitation assays were performed by using the Imprint RNA Immunoprecipitation Kit (Sigma, St. Louis, USA) along with the AGO2 antibody (Cell signaling, Rockford, USA). The AGO2 antibody was then recovered by protein A/G beads. XIST, iASPP and miR-140/miR-124 RNA levels in the immunoprecipitates were measured by qRT-PCR.

### Statistical analysis

Data were exhibited as mean ± SD of three independent experiments and processed using SPSS 17.0 statistical software (SPSS, Chicago, IL, USA). By using Wilcoxon’s paired test we compared the expression of miR-140, miR-124, iASPP and CDK1 in PC tissues and the paired adjacent normal colonic tissues. The differences between groups in other assays were evaluated using the one-way ANOVA. *P* values of <0.05 were considered statistically significant.

## SUPPLEMENTARY MATERIALS TABLE




