# Supplementary material for: The lncRNA XIST interacts with miR-140/miR-124/iASPP axis to promote pancreatic carcinoma growth
Source: Oncotarget. 2017 Nov 20;8(69):113701–18. doi: 10.18632/oncotarget.22555 (PMC5768357; doi:10.18632/oncotarget.22555)
Supplement: Supplementary file 1 [file oncotarget-08-113701-s001.pdf]

The lncRNA XIST interacts with miR-140/miR-124/iASPP axis to promote pancreatic carcinoma growth

SUPPLEMENTARY MATERIALS

Supplementary Table 1: Sequences used in the present study

See Supplementary File 1
