# Supplementary material for: The lncRNA XIST interacts with miR-140/miR-124/iASPP axis to promote pancreatic carcinoma growth
Source: Oncotarget. 2017 Nov 20;8(69):113701–18. doi: 10.18632/oncotarget.22555 (PMC5768357; doi:10.18632/oncotarget.22555)
Supplement: Supplementary file 2 [file oncotarget-08-113701-s002.docx]

**Supplementary Table 1: Sequences used in the present study**

| Name | Sequences |
| --- | --- |
| XIST-F | AGCCTTTCCACCTCTGTC |
| XIST-R | CCTCTTCTGCCACCTGTT |
| sh-XIST-1-F | GATCCATACATTTCTATAATAGTCACCTCGAGGTGACTATTATAGAAATGTATTTTTTG |
| sh-XIST-1-R | AATTCaaaaaATACATTTCTATAATAGTCACCTCGAGGTGACTATTATAGAAATGTATG |
| sh-XIST-2-F | GATCCTTAGTCTTTCCTAACCTTCCACTCGAGTGGAAGGTTAGGAAAGACTAATTTTTG |
| sh-XIST-2-R | AATTCaaaaaTTAGTCTTTCCTAACCTTCCACTCGAGTGGAAGGTTAGGAAAGACTAAG |
| sh-contr-F | ttctccgaacgtgtcacgt |
| sh-contr-R | acgtgacacgttcggagaa |
| miR-124-3P-RT | GTCGTATCCAGTGCAGGGTCCGAGGTATTCGCACTGGATACGACGGCATT |
| miR-124-3P-F | GGCGCG TAAGGCACGCGGTG |
| miR-140-5P-RT | GTCGTATCCAGTGCAGGGTCCGAGGTATTCGCACTGGATACGACCTACCA |
| miR-140-5P-F | GGCGCAGTGGTTTTACCCTA |
| mir-ty-r | GTGCAGGGTCCGAGGT |
| U6-F | CTCGCTTCGGCAGCACA |
| U6-R | AACGCTTCACGAATTTGCGT |
| Wt-XIST-S1-F | AAACTCGAG ataaattatatgtatatt |
| Wt-XIST-S1-R | AAAGCGGCCGC ttcattcattcataaata |
| Mut-XIST-S1-F | atgctgggtgctagagcatggaaagggggaaaagtatt |
| Mut-XIST-S1-R | aatacttttccccctttccatgctctagcacccagcat |
| Wt-XIST-S2-F | AAACTCGAG gcctggcactctagcact |
| Wt-XIST-S2-R | AAAGCGGCCGC ggagacatgaaataaagc |
| Mut-XIST-S2-F | tgctaatttctttcgtgtgacggaatgcctcattttctct |
| Mut-XIST-S2-R | agagaaaatgaggcattccgtcacacgaaagaaattagca |
| Wt-iASPP 3’UTR-F | AAACTCGAG tgacagaaacaagcatt |
| Wt-iASPP 3’UTR-R | AAAGCGGCCGC gactcccaggaatatccaa |
| Mut-iASPP 3’UTR-F1 | gaaatcactggggacaggattggtgttccttttgccaaat |
| Mut-iASPP 3’UTR-R1 | atttggcaaaaggaacaccaatcctgtccccagtgatttc |
| Mut-iASPP 3’UTR-F2 | gaagaaggagggccccagggaataatttagtaatctgcctt |
| Mut-iASPP 3’UTR-R2 | aaggcagattactaaattattccctggggccctccttcttc |
| Si-NC-F | UUCUCCGAACGUGUCACGU |
| Si-NC-R | acgUgacacgUUcggagaa |
| Si-iassp-F | AGACUUUACUCCUUUGAGGCU |
| Si-iassp-R | CCUCAAAGGAGUAAAGUCUAG |
| iassp-F | AAAGGATCCatggacagcgaggcattcca |
| iassp-R | AAAGAATTCctagactttactcctttg |
| P73-F | AAAGGATCCatgctgtacgtcggtgac |
| P73-R | AAA AAGCTTtcagtggatctcggcctc |
| Wt-XIST-PF(promoter) | AAACTCGAGaagcttggctcccttgaggt |
| Wt-XIST-PR(promoter) | AAAAGATCTaagcttccagccccgaga |
| mut-XIST-PF(promoter) | caaatttcttaaaagtatagaacatttccacacttgtaaagtaaacctgtgttcat |
| mut-XIST-PF(promoter) | atgaacacaggtttactttacaagtgtggaaatgttctatacttttaagaaatttg |
| XIST-CF(chip) | gattacaagcatgagcca |
| XIST-CR(chip) | ctaatacaactttatcta |
